# Proximity-dependent biotinylation screening identifies NbHYPK as a novel interacting partner of ATG8 in plants

**DOI:** 10.1186/s12870-019-1930-8

**Published:** 2019-07-19

**Authors:** Mercy W. Macharia, Wilfred Y. Z. Tan, Prem P. Das, Naweed I. Naqvi, Sek-Man Wong

**Affiliations:** 1 Department of Biological Sciences, National University of Singapore, Singapore, 119543 Singapore; 2 Temasek Life Sciences Laboratory, Singapore, 117604 Singapore; 3 National University of Singapore Research Institute, Suzhou, Jiangsu 215123 People’s Republic of China

**Keywords:** BioID, Autophagy, ATG8, TMV, TMV 24A + UPD, Protein-protein interaction, HYPK

## Abstract

**Background:**

Autophagy is a conserved, highly-regulated catabolic process that plays important roles in growth, development and innate immunity in plants. In this study, we compared the rate of autophagy induction in *Nicotiana benthamiana* plants infected with Tobacco mosaic virus or the TMV 24A + UPD mutant variant, which replicates at a faster rate and induces more severe symptoms. Using a BirA* tag and proximity-dependent biotin identification (BioID) analysis, we identified host proteins that interact with the core autophagy protein, ATG8 in TMV 24A + UPD infected plants. By combining the use of a fast replicating TMV mutant and an in vivo protein-protein screening technique, we were able to gain functional insight into the role of autophagy in a compatible virus-host interaction.

**Results:**

Our study revealed an increased autophagic flux induced by TMV 24A + UPD, as compared to TMV in *N. benthamiana*. Analysis of the functional proteome associated with ATG8 revealed a total of 67 proteins, 16 of which are known to interact with ATG8 or its orthologs in mammalian and yeast systems. The interacting proteins were categorized into four functional groups: immune system process, response to ROS, sulphur amino acid metabolism and calcium signalling. Due to the presence of an ubiquitin-associated (UBA) domain, which is demonstrated to interact with ATG8, the Huntingtin-interacting protein K-like (HYPK) was selected for validation of the physical interaction and function. We used yeast two hybrid (Y2H), bimolecular fluorescence complementation (BiFC) and subcellular localization to validate the ATG8-HYPK interaction. Subsequent down-regulation of ATG8 by virus-induced gene silencing (VIGS) showed enhanced TMV symptoms, suggesting a protective role for autophagy during TMV 24A + UPD infection.

**Conclusion:**

This study presents the use of BioID as a suitable method for screening ATG8 interacting proteins *in planta*. We have identified many putative binding partners of ATG8 during TMV 24A + UPD infection in *N. benthamiana* plants. In addition, we have verified that NbHYPK is an interacting partner of ATG8. We infer that autophagy plays a protective role in TMV 24A + UPD infected plants.

**Electronic supplementary material:**

The online version of this article (10.1186/s12870-019-1930-8) contains supplementary material, which is available to authorized users.

## Background

Induction of autophagic vesicles has been reported with infection of several viruses in both plant and animal cells [1–4]. Unless impaired, autophagy pathways endeavour to actively eliminate intracellular invaders to promote cell survival [2]. Studies have revealed an important role of autophagy in plant innate immunity, programme cell death, plant resistance and viral RNA silencing [4–6]. Recent studies have continued to elucidate roles of autophagy, its basic mechanism and interaction with other host components during virus infection in plants. ATG8 directly interacts and facilitates elimination of *Cotton leaf curl Multan virus* (CLCuMuV) virulence factor [5] and *Cauliflower mosaic virus* (CaMV) capsid protein P4 [6]. Calmodulin-like protein NbCaM, interacts with *N. benthamiana* Suppressor of Gene Silencing 3 (NbSGS3) and promotes autophagic degradation during geminivirus infection [7]. During *Turnip mosaic virus* (TuMV) infection, selective autophagy cargo receptor NBR1 targets the RNA silencing suppressor HCpro, thus suppressing viral accumulation [8]. ATG6 interacts with TuMV RNA-dependent RNA polymerase (RdRp) and restricts its infection [9].

RNA viruses exploit cellular machinery into virus assembly complexes through various compositions of protein-RNA, protein-lipid and protein-protein components [10]. Their interaction with plant autophagy is largely unexploited. Therefore, there is a need to identify major host proteins that are involved in autophagy during compatible RNA virus-host interaction. Autophagy induces the formation of autophagosomes which are double-membrane vesicles that engulf cargoes such as proteins, damaged organelles and pathogens and transport them to the vacuoles for degradation [11]. ATG8 is a core protein associated with maturation of the autophagosome [12]. Therefore, screening for ATG8 interacting proteins is crucial in understanding the basic mechanism of autophagosome formation and processing during viral infection.

*Tobacco mosaic virus* (TMV) is a positive sense RNA virus that is compatible with certain genotypes of tobacco plants. It has an upstream pseudo-knot domain (UPD) at the 3′ end that plays a crucial role during virus translation and expression [13]. TMV 24A + UPD, a mutant engineered by introducing an internal poly (A) tract (24 nucleotides) upstream of the TMV UPD induced earlier and more severe necrotic symptoms [14]. Using this mutant, we investigated autophagy induction and flux in infected *N. benthamiana* plants. We screened for host proteins that interact with ATG8 during mutant virus infection *in planta*. In this report, we showed that TMV 24A + UPD, a faster replicating mutant, enhanced autophagy induction and flux, as compared to TMV. We employed the BioID technique by fusing the biotin ligase BirA* to ATG8. The technique was initially used in mammalian cells [15] and more recently used in rice protoplasts [16] and detached leaf tissues [17]. The BirA* releases biotinyl-5′-AMP which chemically attaches biotin to proteins that are located within 10 nm of its proximity. Subsequently, proteins that are close to or interacting with the target protein ATG8 are biotinylated. They can be purified using streptavidin and analysed by tandem mass spectrometry (MS/MS).

A total of 67 proteins were identified, most of which are previously unknown to interact with autophagy proteins in plants. Sixteen of the proteins have been previously shown to directly or indirectly associate with autophagy pathway in animal and/or yeast system. Due to the presence of the UBA domain, which is known to interact with ATG8, the Huntingtin-interacting protein K-like (HYPK) was selected for further validation. Using yeast-two-hybrid (Y2H), biomolecular fluorescence complementation (BiFC) and subcellular co-localization, we showed that HYPK and ATG8 interacted in *N. benthamiana* plants. Knockdown of NbHYPK did not enhance viral symptoms, in contrast to the observed decrease in symptoms upon autophagy knockdown, suggesting that NbHYPK alone does not influence viral symptoms in test plants. The full length primary sequences of mammalian HYPK and NbHYPK-like proteins share a 57% similarity of conserved and similar amino acids. Both of the proteins have a conserved UBA domain at the C terminus. This shared similarity suggests that NbHYPK may play a role similar to the mammalian ortholog. The mammalian HYPK is an intrinsically unstructured chaperone that interacts with Huntingtin (HTT) and it enhances autophagy flux and increases in conversion of ATG8 to the lipidated/active ATG8-PE (LC3I to LC3II) [18].

## Results

### TMV 24A + UPD induced autophagosome biogenesis and autophagic flux

**Fig. 1:**
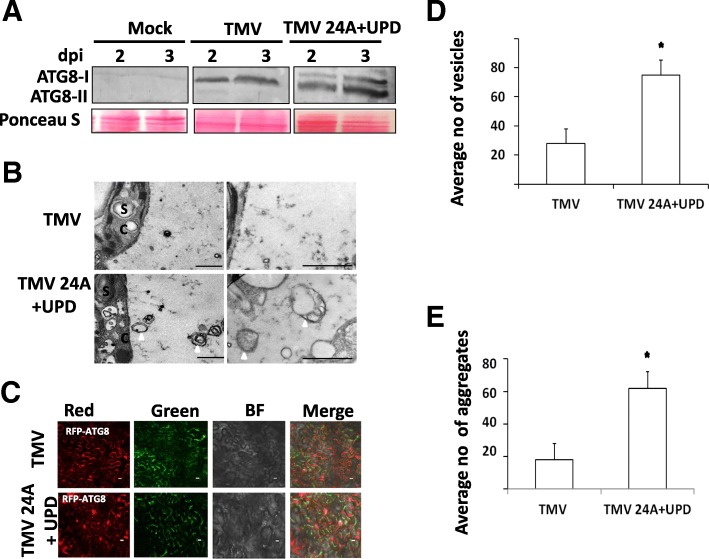
Autophagy activation in response to TMV and TMV24A + UPD infection in *N. benthamiana****.*** **a** Immunoblot analysis using ATG8 antibody. Molecular weight of the bands detected at ~ 16 kDa. The Ponceau S staining served as a loading control (lower panel, Ponceau S). **b** Representative electron micrographs showing infected plants at 3 dpi (days post inoculation). Arrows show membranous autophagosome-like structures. Abbreviations; S, starch, C, cytoplasm. **c** Representative images of RFP-ATG8 labelled autophagosomes at 3 dpi. **d** The number of autophagosome-like structures was obtained from ~ 100μm^2^ section for each experiment. Autophagosome-like structures were significantly enhanced by infection with TMV 24A + UPD (*p < 0.05). **e** The mean number of fluorescent puncta/aggregates were obtained from sections with approximately equal number of cells (~ 100) for each experiment. RFP-ATG8a autophagosomes were significantly enhanced by infection with TMV24A + UPD (*p < 0.05). The asterisks indicate significant differences by unpaired sample *t* test. The scale bar represents 1 μm

TMV 24A + UPD replicated faster and induced more severe symptoms, as compared to TMV [14]. We hypothesized that rapid virus replication could increase autophagy and/or autophagic flux. To determine whether autophagy is triggered upon infection by TMV and TMV 24A + UPD, we detected autophagic flux by confirming the presence of ATG8-II at 2 and 3 dpi (Fig. 1a; Additional file 1: Figure S1). Next, we investigated the ultrastructural changes in infected leaves by transmission electron microscopy (TEM). Membranous vesicles resembling autophagosome-like structures were observed in ultrathin sections of TMV 24A + UPD infected leaves (Fig. 1b). To verify autophagy induction, we infiltrated RFP-ATG8 into virus-infected *N. benthamiana* leaves, and quantified the number of epifluorescent foci (Fig. 1c).

The number of autophagosome-like structures in TMV 24A + UPD infected plants were significantly higher than those in TMV infected plants (Fig. 1d). A significantly higher number of RFP-ATG8 labelled aggregate structures were observed in TMV 24A + UPD, as compared to that of TMV (Fig. 1e).

### Identification of biotinylated proteins

**Fig. 2:**
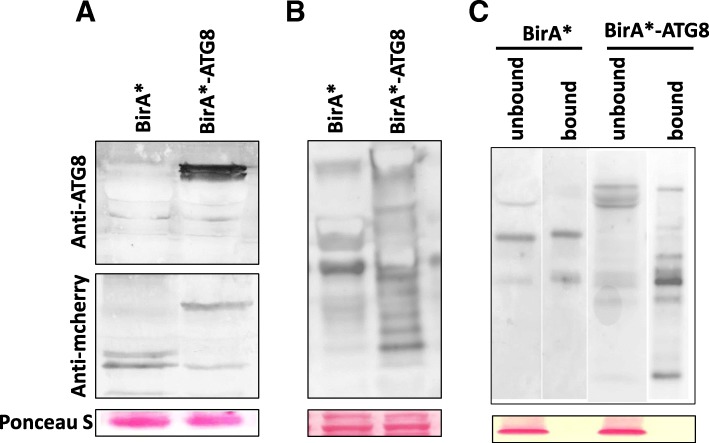
Immunoblot analysis of BioID fusion proteins and total biotinylated proteins. **a** Western blots were carried out using anti-ATG8, or anti-mCherry antisera to ascertain fusion protein expression. **b** Streptavidin-HRP blot of crude protein lysate. **c** Strep-HRP blot after Dynabeads™ purification. Lower panels show Ponceau S stained blot membranes to confirm equal loading of total proteins

After determining that TMV 24A + UPD induced autophagic flux, we sought to identify ATG8 interacting proteins during infection. Two plasmids, PXY01-RFP-HA-BirA* (BirA*) used as a control and PXY01-RFP-HA-BirA*-ATG8 (BirA*-ATG8) used as baits, were constructed (Additional file 1: Figure S2a). Expression of BirA* and BirA*-ATG8 were successful for both constructs (Fig. 2a; Additional file 1: Figure S2b, 2c, 2d, 2e). *N. benthamiana* leaves were agroinfiltrated either with BirA* or BirA*-ATG8 vectors, with agroinfiltration buffer supplemented with 2 mM biotin. Biotinylated proteins were detected by Western blot and purified using Magnetic Dynabeads™ coated with streptavidin (Fig. 2b, c; Additional file 1: Figure S3a, b). The BirA*-ATG8 bound fraction showed multiple biotinylated protein bands (Fig. 2c; Additional file 1: Figure S3c). This suggests that there were more biotinylated proteins in the beads fraction of BirA*-ATG8 samples, as compared to the control.

### ATG8 potentially interacts with a network of proteins involved in plant defense

**Fig. 3:**
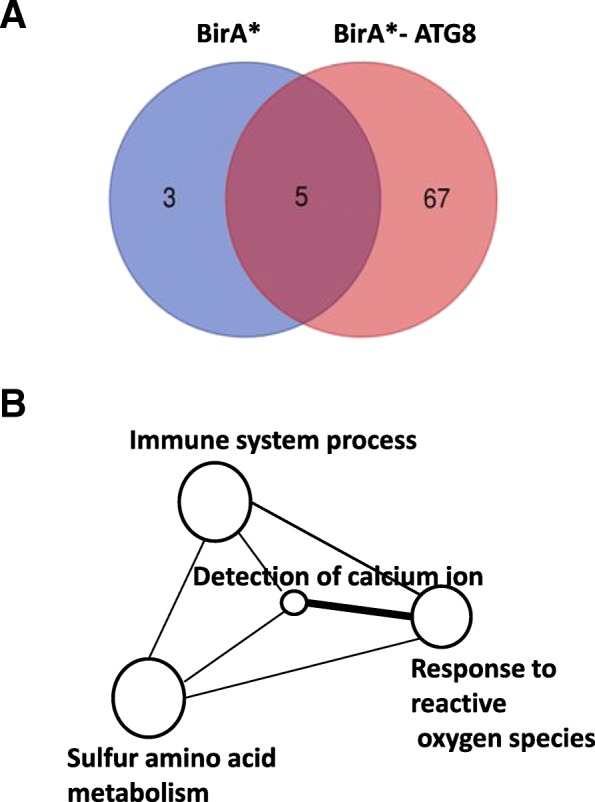
Statistics of proteins identified and Gene ontology (GO) enrichment analysis of unique BirA*-ATG8 proteins. **a**Venn diagrams showing unique and shared proteins between BirA* and BirA*-ATG8. **b** Interactive graphs of proteins that were identified with BirA*-ATG8 during TMV 24A + UPD infection. The sizes of the circles are proportional to the number of proteins associated with the specific term. The interactive network were summarized and plotted following published REVIGO protocol (http://revigo.irb.hr/)

Subsequent bioinformatics analysis of MS/MS results revealed 5 proteins that were shared between the BirA* and BirA*-ATG8 extracts and 67 proteins were unique to BirA*-ATG8 (Fig. 3a). Gene ontology analysis categorized BirA*-ATG8-identified proteins into 4 functional groups namely: immune system process, response to ROS, sulphur amino acid metabolism and calcium ion signalling (Fig. 3b). Of these proteins, 14 were uncharacterized and 23 were not reported to be involved in autophagy or plant defense (Additional file 2: Table S1). Therefore, they were not selected as candidates for further validation. The rest of the proteins identified were deemed significant, based on published literature (Table 1). These proteins may interact directly or indirectly with ATG8 in *N. benthamiana* plants. The first 16 proteins have been previously shown to interact with the autophagy pathway (Table 1). Proteins 17 to 30 have not been shown to interact with autophagy processes but have biological roles in plant defense and may likely interact with the core autophagy components. We selected the HYPK protein for validation of ATG8 interaction because it contains a UBA domain which has been demonstrated to interact with ATG8.

**Table 1:** BirA*-ATG8 associated proteins identified via BioID

No.	Accession	Protein name
1.	I7GVS5	Heat shock protein 70 (Hsp70) [18, 19] .
2	A2PYH3	Alpha chain of nascent polypeptide associated complex (NAC) [20].
3	A0A1J6JLR9	Eukaryotic translation initiation factor 4 g [21].
4	A0A1P8SF07	TOM1-like protein 2 (Tom1L2) [22]
5	Q4QXL9	Autophagy-related Protein 3 (ATG3) [23, 24].
6	A0A1U7YMI0	Glyceraldehyde-3-phosphate dehydrogenase (GAPDH) [24].
7	A0A1S3ZA42	FK506-binding protein 5 (FKBP5)-like isoform X2 [25].
8	A0A1U7XHY8	Subtilisin-like protease (SLP) [26].
9	Q8W183	Carbonic anhydrase [27].
10	Q76MF3	Calmodulin [28].
11	A0A1U7YG19	Serine hydroxymethyltransferase (SHMT) [29].
12	A0A1U7VJK4	Plasminogen activator inhibitor 1 (PAI-1) RNA-binding protein-like [30].
13	A8UDS9	Copper/zinc superoxide dismutase (SOD1) [31].
14	A0A1U7XPN3	Huntingtin-interacting protein K (HYPK) [32] .
15	A0A1S3XIE4	Acyl-CoA-binding domain-containing protein 3-like [33].
16	A0A1U7XC68	Clathrin light chain [34].
17	A0A1P8SF08	AvrPto-interacting protein 1 (Api1)
18	A0A1J6K7H0	Elongation factor 1-alpha (eEF1A)
19	A0A1U7XRQ0	H/ACA ribonucleoprotein (RNP) complex subunit 1-like isoform X2 [35].
20	Q9SXX4	Fructose-bisphosphate aldolase [36]
21	A0A1U7YCY5	Epidermal growth factor receptor substrate 15 (EPS15)-like 1
22	Q6K0Q3	Chloroplast glutamine synthetase (GS) [37, 38].
23	C9DFB0	ASR
24	Q8LKF9	SGT1-like protein
25	A0A1U7YZQ4	probable ADP-ribosylation factor GTPase-activating protein (Arf GAP) AGD6
26	A0A1U7YFQ9	ATP-dependent RNA helicase SUPV3L1, mitochondrial-like
27	A0A1U7XQL2	40S ribosomal protein S20–2 (RPS20)
28	A0A1U7WRW2	40S ribosomal protein S6 (RPS6) [39]
29	A0A1U7VRZ1	Charged multi-vesicular body protein 5 (CHMP5)-like
30	A0A1U7VDD8	Actin cytoskeleton-regulatory complex protein PAN1-like isoform X1 [40].

### NbHYPK interacts with ATG8

**Fig. 4:**
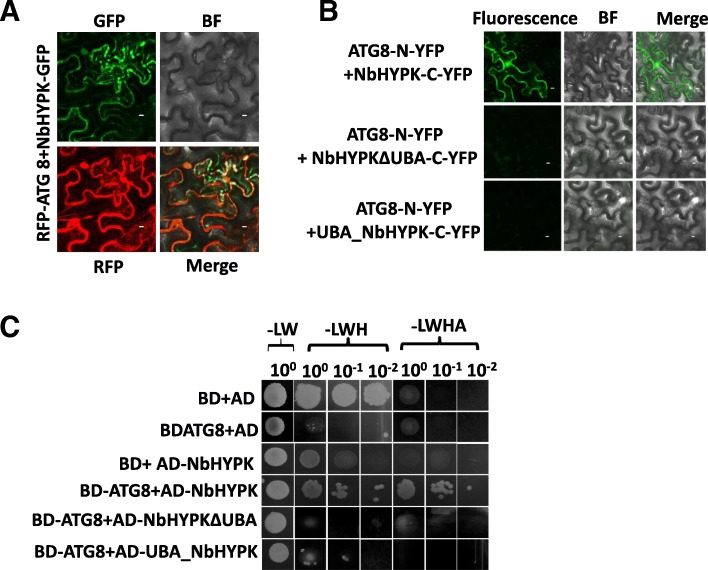
ATG8 directly interacts with NbHYPK**. a** GFP labelled NbHYPK, NbHYPKΔUBA and UBA-NbHYPK was co-infiltrated with RFP-labelled ATG8 and localized by confocal microscopy. **b** Bimolecular fluorescence complementation (BiFC) analysis, ATG8-Yn or x-Yn(empty vector) was co-expressed with NbHYPK-Yc, NbHYPKΔUBA-Yc and UBA-NbHYPK-Yc or x-Yc (empty vector). Fluorescent signals were visualized after 3 dpi by confocal microscopy. Bar = 1 μm. **c** Analysis of interaction between ATG8 and NbHYPK by Yeast two hybrid assay. Plasmids expression full length NbHYPK or the UBA domain or NbHYPKΔUBA or empty vector were transformed into AH109 yeast and mated with yeast expressing plasmids containing ATG8 or an empty vector and grown in amino acid drop out YPDA media

NbHYPK was identified from BioID screening and verified by cDNA cloning and sequencing. NbHYPK is a small protein spanning 109 amino acids. It has a UBA domain between amino acids 68–109 and is denoted as UBA_NbHYPK. In order to test if the UBA domain is required for its interaction with ATG8, it was deleted from the NbHYPK gene and referred to as NbHYPKΔUBA (1–67 aa). We confirmed expression of full length and truncated fusion proteins of HYPK-GFP and RFP-ATG8 by Western blotting (Additional file 1: Figure S4a). We then conducted a co-localization assay After NbHYPK-GFP / NbHYPKΔUBA-GFP/ UBA_NbHYPK-GFP and mCherry-ATG8 were co-infiltrated into *N. benthamiana* leaves and transiently expressed, aggregates were observed only in leaves co-expressing NbHYPK-GFP and mCherry-ATG8 (Fig. 4a), but with no aggregates observed in leaves expressing NbHYPKΔUBA or UBA_NbHYPK-GFP and mCherry-ATG8 (Additional file 1: Figure S4b). To further confirm the interaction between NbHYPK and ATG8, we performed a BiFC assay. Constructs with NbHYPK fused to the C-terminus of yellow fluorescent protein (NbHYPK -Yc) and ATG8 fused to the N-terminus of YFP (ATG8-Yn) were co-expressed in *N. benthamiana* leaves and resulted in reconstitution of YFP (Fig. 4b). Leaves expressing ATG8-Yn with UBA_NbHYPK –Yc, NbHYPKΔUBA -Yc or other empty vector controls failed to generate epifluorescent signals (Additional file 1: Figure S4c).

Additionally, we employed Y2H to confirm the interaction between NbHYPK and ATG8. ATG8 was fused to the binding domain (BD) and tested for its interaction with NbHYPK fused to the activation domain (AD). Y2H assays validated the physical interaction between NbHYPK and ATG8 (Fig. 4c). We also constructed two other plasmids, NbHYPKΔUBA and UBA_NbHYPK, into the AD and tested for their interactions with BD-ATG8. No interaction was evident (Fig. 4c). Taken together, we conclude that the newly identified host protein NbHYPK physically interacts with ATG8 in vitro and in vivo. In addition, we demonstrated that the UBA domain in NbHYPK is essential for such physical interaction with ATG8.

### Autophagy protects *N. benthamiana* plants against rapid cell death induced by TMV 24A + UPD

**Fig. 5:**
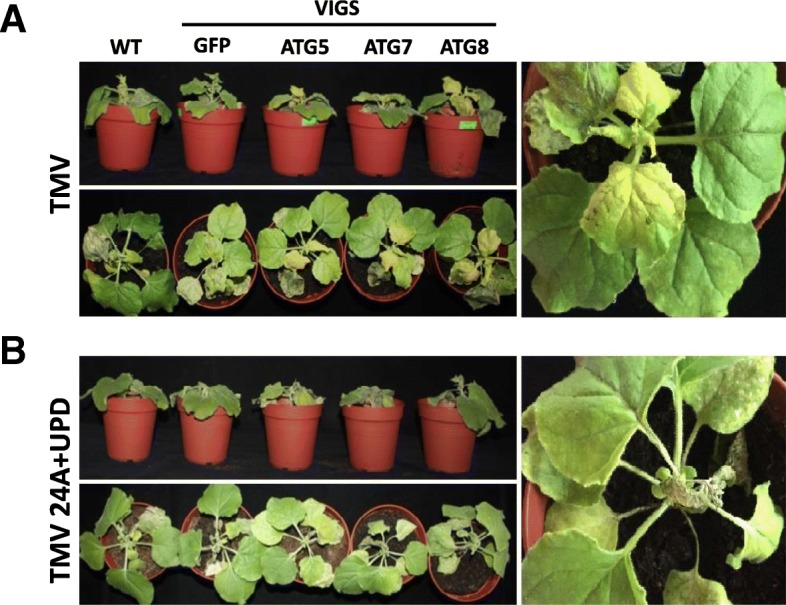
Silencing important gene(s) essential for autophagy, accelerated TMV 24A + UPD cell death symptoms. **a**TMV infected plants showing chlorosis in autophagy silenced plants. Inset is an enlarged section of ATG7-silenced plants. **b** TMV 24A + UPD showing accelerated cell death in autophagy silenced plants. An enlarged ATG7 silenced plant showing accelerated cell death in the emerged leaves

**Fig. 6:**
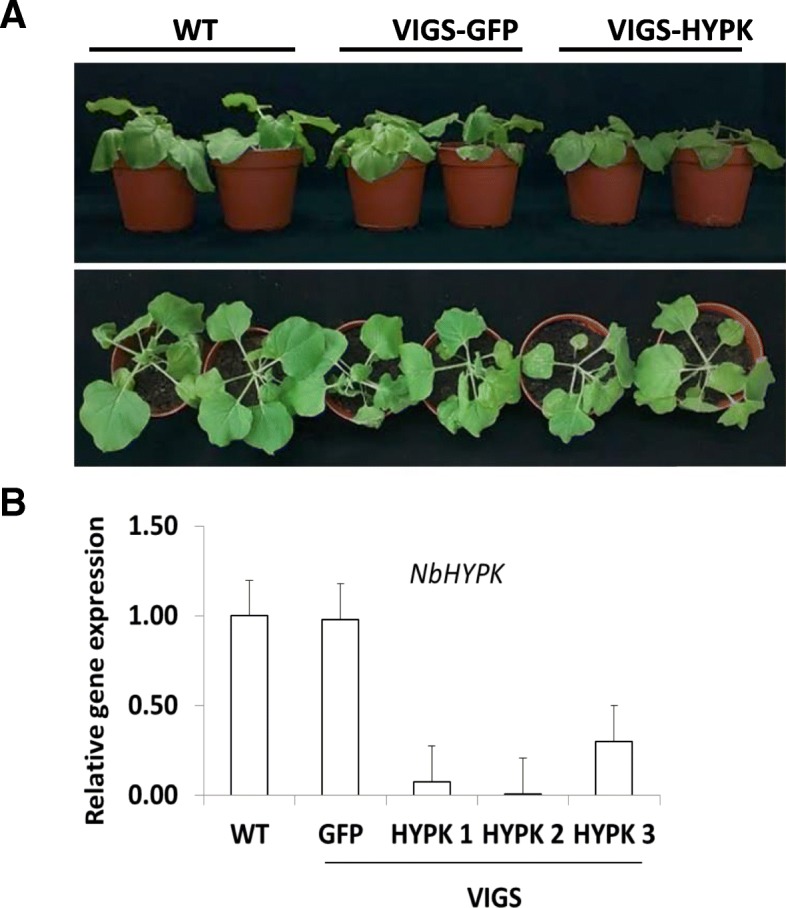
TMV 24A + UPD symptoms and gene expression analysis in VIGS-NbHYPK plants**. a** VIGS-silenced plants showed no significant difference in viral symptoms as compared to non-silenced plants. **b** Quantitative real-time (qrtPCR) analysis of NbHYPK gene in control and VIGS-NbHYPK plants

To test whether autophagy was required for TMV 24A + UPD induced cell death, we silenced ATG5, ATG7 and ATG8, which are core components of autophagy. Such autophagy silenced plants infected with TMV showed increased chlorosis but did not show severe cell death symptoms (Fig. 5a). Cell death triggered by TMV 24A + UPD was markedly increased in ATG5 and ATG7 silenced plants (Fig. 5b). Despite targeting the conserved regions of ATG8 isoforms, ATG8 silencing was less efficient, as compared to ATG5 and ATG7 in both TMV and TMV 24A + UPD infected plants (Additional file 1: Figure S5a, b). Perhaps transient ATG8 silencing was less efficient as ATG8 has 8 isoforms in *N. benthamiana* [41]. The silenced TMV 24A + UPD infected plants showed enhanced cell death symptoms in the newly emerged leaves (Fig. 5b). We infer that autophagy plays a protective role in TMV 24A + UPD infected plants. NbHYPK-silenced plants did show slight but not any obvious differences in viral symptoms, as compared to the normal NbHYPK expressing plants (Fig. 6a). No significant difference was found in coat protein gene expression between NbHYPK-silenced and control plants (Additional file 1: Figure S6a), as quantified by ImageJ [42]).

## Discussion

Several studies have defined autophagy in yeast and mammalian systems [25, 29]. However, autophagy pathways in plants have not been elucidated extensively. In this study, we compared the autophagy induction and flux by TMV and a fast replicating mutant TMV 24A + UPD in *N. benthamiana* plants. An increase of autophagy flux was detected at 2 and 3 dpi (Fig. 1a). Significantly higher number of autophagosome-like structures were detected in plants infected with the faster replicating TMV 24A + UPD variant (Fig. 1b - e). This indicates that induction of autophagy is likely accelerated by increased viral load due to a more rapidly replicating TMV virus.

Out of the 67 putative ATG8 interacting proteins identified through BioID screening, 16 proteins were directly associated with autophagy pathways (Table 1). Among these, ATG3 and calmodulin have been shown to interact directly with ATG8 and autophagosomes in *N. benthamiana* plants [7, 23]. A subtilisin-like protease implicated in programmed cell death and defense signaling in plants, plays a role in animal autophagy [25, 41, 42]. Acyl-CoA-binding domain-containing protein 3-like has been shown to disrupt autophagosome formation and increase ATG8 degradation in plants [33]. Most of the other proteins identified in this group are involved with autophagy in mammalian systems but have not been reported in plants. On the other hand, proteins 17 to 30 (Table 1) are involved in autophagy-associated functions such as pathogen defense, stress signalling, and hypersensitive response (HR) [4, 42, 43]. Proteins 1 to 24 (Additional file 2: Table S1) have biological functions that are not associated with autophagy. Recent studies have revealed autophagy-independent functions of ATG8 in maintaining lipid droplet integrity in yeast and in apicoplast formation in malaria [44–46]. Perhaps ATG8 isoform(s) also play an autophagy-independent role in plants. Some proteins that interact with ATG8, such as ATG4 and ATG7, were not identified through MS/MS. Some autophagy proteins may be difficult to purify as they unstable [47]. In addition, some proteins not interacting with ATG8 were also biotinylated due to their incidental proximity to the BirA*-ATG8 fusion protein in cells, an inherent limitation of the BioID technique. Therefore, interaction studies such as Y2H, BiFC and co-localization were further considered to confirm the physical interactions.

High throughput protein-protein interaction studies are crucial in narrowing down candidates to study autophagy. ATG8 interacting proteins typically possess an AIM/LIR motif. While this motif is used to select candidates, it does not always represent functionality of the motif [48]. Therefore, additional criteria are employed, such as structural and biological aspects of the proteins. Autophagy receptors Joka2, P62 and NBR1 have UBA-like domain(s) that are crucial in their interaction with ATG8 [49]. Analysis of the ATG8 interactor NbHYPK (this study) revealed that the primary sequence does not contain a conventional AIM motif. However, it contained a UBA domain in the C-terminus. Thus, we opted to further validate NbHYPK interaction with ATG8 using the aforementioned analyses.

Our results showed that NbHYPK and NbATG8 interacted physically. The interaction of NbHYPK and ATG8 requires the UBA domain. The UBA domain of NbHYPK has high similarity with another cytosolic chaperone, nascent polypeptide-associated complex proteins (NAC), which is associated with post translational processes of nascent proteins emerging from the ribosomes [50]. We also identified the alpha chain of nascent polypeptide associated complex protein (Table 1). The mammalian Plic-2 protein possesses a UBA domain and it is involved in autophagy [51]. Plic-2 has played a protective role during starvation and requires ATG5 and ATG7 proteins. It directly interacts with ATG8 (LC3) and plays a role in fusion of autophagosomes to lysosomes [51].

Although down-regulation of NbHYPK using VIGS produced no significant difference in viral symptoms and cell death in NbHYPK-silenced plants, silencing NbHYPK may not produce phenotypes but may have an effect on other cellular processes, as evidenced by the mammalian HYPK ortholog. The mammalian huntingtin-interacting protein K (HYPK) has chaperone-like properties and is involved in cell death, unfolded protein response, cell cycle and cell growth [52]. Overexpressing HYPK increased conversion of the mammalian homologues of ATG8 and ATG8-PE, LC3I to LC3II, respectively [18]. Interestingly, the Huntingtin protein itself (HTT) has been shown to interact with ATG8 and is important for autophagy in Drosophila [32]. We do not rule out the redundancy in chaperone functions in targeting ATG8.

On the other hand, knockdown of ATG5, ATG7 and ATG8 revealed that autophagy plays a protective role against TMV 24A + UPD induced cell death. Although autophagy seems to serve as an antiviral mechanism against TMV, it is still intriguing that there is no significant autophagic flux, as compared to TMV 24A + UPD. Autophagy has been shown to play an antiviral role [8], thus, it is possible that TMV subverts autophagy by an as yet unknown mechanism. A recent study has shown that *Barley stripe mosaic virus* (BSMV) interrupts autophagy by disrupting the interaction between ATG8 and ATG7 [47]. It is also plausible that the induction of autophagy flux by TMV 24A + UPD is a result of an altered host-pathogen interaction. Reactive oxygen species (ROS) can induce autophagy in mammalian and plant cells [53, 54]. Autophagy plays a significant role to alleviate oxidative damage by reducing ROS [53, 54]. Future studies will aim at identifying whether early infection by TMV 24A + UPD elicits an increased ROS which likely results in accelerated cell death.

## Conclusion

Using the BioID technique, we have identified 67 proteins that interact with ATG8 in *N. benthamiana* plants infected with TMV and its more rapidly replicating mutant TMV 24A + UPD. A new ATG8 interacting protein, NbHYPK, was identified and verified for its interaction with ATG8. The interaction requires the presence of the UBA domain in NbHYPK. A recent study has described a new class of ATG8 adapters and receptors that exploit ubiquitin-interacting motif sequences for high-affinity binding [55].

In summary, NbHYPK is involved in and may regulate autophagic processes by ATG8 during viral/TMV infection. Future studies will focus on elucidating the biological significance of NbHYPK chaperone activity and its relationship with other novel interacting partners identified in the functional proteome associated with ATG8 in *N. benthamiana* plants.

## Methods

### Plant materials, plasmid construction and agrobacterium infiltration

*Nicotiana benthamiana* seeds were obtained from Professor Dawei Li of China Agricultural University, Beijing, PRC. Seedlings and plants were grown in pots placed in growth rooms at 25 °C under a 16-h-light/8-h-dark cycle. NbATG8a (referred to as ATG8) was amplified using primers in Additional file 3: Table S2. The cDNA clone was used to construct vector PXY01 -35S-mCherry-ATG8a. The construct was transformed into Agrobacterium. For VIGS experiment, ATG5, ATG7 and ATG8 targets were amplified using primers listed in Additional file 3: Table S2. The resulting fragments were inserted into pTRV2. Together, pTRV1 and pTRV2 were introduced into Agrobacterium as previously described [56]. Down-regulation of the gene expression for NbHYPK, ATG5, ATG7 and ATG8 was confirmed by qRT-PCR/RT-PCR (Fig. 6b; Additional file 1: Figure S5, S6b). Cloning was done using either ligation of restriction enzyme generated sticky ends (enzyme sites underlined) or Gibson Assembly® cloning kit from NEB kit, see primers (prefix GA is used) in Additional file 3: Table S2.

*E. coli* biotin ligase BirA* was amplified from pcDNA 3.1 myc BioID plasmid using appropriate primers (Additional file 3: Table S2). Xba1 RE site and HA tag sequences were included in the forward primer while Sma1 was included in the reverse primer. The resulting fragment was digested with appropriate enzymes and inserted into vector PXY0 -35S-mCherry-ATG8. The resulting vector PXY01-mCherry-HABirA*-ATG8 (BirA*-ATG8) was confirmed by DNA sequencing. For the control plasmid, the fragment was inserted into PXY01-mCherry-X and resulted into PXY01-mCherry-HABirA* (BirA*). BioID clones were transformed into agrobacterium, and 2.5 mM biotin incorporated into the agroinfiltration buffer. The clones were agroinfiltrated into fully expanded leaves and 2.5 μg in vitro transcribed TMV 24A + UPD viral RNA was mechanically inoculated into the plants. The leaves were collected at 3dpi and total proteins extracted. Protein expression was verified by immunoblot using mCherry and ATG8 antibodies. For BiFC and subcellular localization, PCR fragments were amplified with primers shown in Additional file 3: Table S2 and cloned into respective vectors. BiFC vectors were transformed into Agrobacterium. The two cultures, nYFP-ATG8 and cYFP –NbHYPK, were mixed at a ratio of 1:1 and co-inoculated into *N. benthamiana* plants as previously described [28]. For subcellular co-localization, NbHYPK-GFP and RFP-ATG8 vectors were also co-cultured as above.

### Western blotting

Total proteins were extracted from *N. benthamiana* leaves and separated by SDS-PAGE for immunoblot analysis with appropriate antibody. After proteins were transferred to nitrocellulose membrane, Ponceau S was used to stain proteins for loading control, according to manufacturer’s protocol, before proceeding with blocking and antibody incubation. Protein gel band images were captured and converted into JPEG format and subjected to digital image analysis using ImageJ software version 1.52a [42].

### Confocal microscopy

For autophagosome visualization, fully expanded leaves were agroinfiltrated with RFP-ATG8. After 24 h, the infiltrated leaves were inoculated mechanically with 2.5 μg in vitro transcribed RNA in GKP buffer (50 mM glycine; 30 mM K_2_HPO_4_, pH 9.2; 1% bentonite; 1% celite). After 3 days, leaves were syringe-infiltrated with 5 uM of autophagy inhibitor E64d for 12 h, immediately followed by confocal microscopy. For subcellular localization and BiFC, leaves were observed for fluorescence 3 days after agroinfiltration.

Live cell images were acquired from abaxial leaf epidermal cells using a Zeiss LSM510 microscope. GFP and RFP probes were excited at 488 and 561 nm, respectively, and their fluorescent emissions detected at 495–550 and 570–610 nm, respectively. YFP was excited at 514 nm with an emission of 527 nm. Confocal images were processed with ZEN (version 2011) and 578 Image J (version 1.48v) software.

### Tem

Virus infected and/or autophagy inhibitor treated leaf samples were fixed in fixation medium (2% paraformaldehyde (w/v), 2.5% glutaraldehyde (v/v) in phosphate buffer (0.1 M, pH 7.0) overnight and post-fixed with 1% OsO_4_ in the phosphate buffer for 2 h. After a series of ethanol dehydration, leaf samples were transferred to acetone for 20 min and then embedded in Spurr® resin. The resin blocks were trimmed and ultrathin sectioned. After staining with 2% uranyl acetate and 1% alkaline lead citrate, the ultrathin sections were observed under a TEM (model JEOL JEM-1230).

### Yeast two hybrid (Y2H) assays

The genes of interest (Additional file 3: Table S2) were cloned into either PGADT7 (AD) or PGBKT7 (BD) yeast plasmid. The recombinant vectors were transformed into AH109 yeast using lithium chloride as described by Matchmaker® Yeast 2 Hybrid protocol. Transformed yeast colonies were grown in liquid media and diluted into 10-, 100- and 1,000-fold and spread over on SD/−Trp/−Leu, SD/−His/−Trp/−Leu and SD/−His/−Trp/−Leu/−Ade plates. The plates were incubated at 30 °C until colonies appeared 3 days later. Colonies that formed on more stringent selection using SD/−His/−Trp/−Leu and SD/−His/−Trp/−Leu/−Ade plates indicated physical interaction between the bait and prey proteins.

### BioID sample preparation and MS/MS analysis

The protein lysate was subjected to phenol extraction. The resulting pellet was dissolved in lysis buffer (0.4% SDS, 500 mM Nacl, 5 mM EDTA, 1 mM DTT, 50 mM Tris-HCL, pH 7.4). Protein concentration was determined using Bradford assay. Streptavidin-coated Dynabeads (200 μl) was gently added into 100 μg of total proteins. The mixture was incubated at 4 °C overnight with gentle shaking. The beads containing bound biotinylated proteins were separated from unbound proteins using a magnetic stand. An aliquot of the unbound proteins was saved for Western blot.

The beads were washed several times (in 1 ml of 1 X PBS per wash with gently shaking, 5 min at room temperature), once in 2% SDS, once in 1% SDS, twice in 1% NP-40 and twice with 1 X PBS. The beads were left in the final wash. An aliquot of the beads was saved for Western blot analysis using streptavidin antibody (1:2000). The beads were then subjected to MS analysis. Briefly, the beads were resuspended in 200 μl triethylammonium bicarbonate.

(TEAB) (0.5 M, pH 8.5) with 4 μl Tris(2-carboxyethyl)phosphine (TCEP) (100 mM stock) by gently mixing at 65 °C for 1 h. Four μl methyl methanethiosulfonate (MMTS) (200 mM stock) was added into the mixture above and incubated at room temperature for 15 min. Tryspin was added into the beads at 12.5 ng/μl and incubated at 37 °C for 16 h with gentle shaking. The beads were pelleted and the supernatant subjected to MS analysis. MS/MS data was searched against database of *Nicotiana* proteins using ProteinPilot™. Data cleaning was carried out to remove insignificant hits. BirA*-ATG8 proteins that overlapped with the control BirA* proteins were also removed. Redundant and low quality proteins were excluded from the final analysis. The proteins were functionally classified. Gene ontology interactive network were summarized and plotted following published REVIGO protocol (http://revigo.irb.hr/ diagrams were generated using UGen Venn software (http://bioinformatics.psb.ugent.be/webtools/Venn/).

## Additional files

Additional file 1: **Figure S1.** Full immunoblot images for Fig. 1a using ATG8 antibody. (a) Mock. (b) TMV. (c) TMV 24A + UPD. **Figure S2.** Expression of BirA* and BirA_ATG8 constructs. **(**a) Graphical representation of control BirA*and experimental plasmids BirA*-ATG8. Full gel image for Fig. 2a carried out using anti-mCherry antiseri, for both (b) & (c) and anti-ATG8 antiserum (* indicates a weak band). (d) to ascertain fusion protein expression. (e) Detection of RFP signals from agroinfiltrated *N.benthamiana* leaves to confirm expression of RFP-fused BirA* and BirA*-ATG8. **Figure S3.** Full gel Immunoblot analysis of total biotinylated proteins for Fig. 2b and c. (a) Streptavidin-HRP blot of crude protein lysate. (b)Strep-HRP blot after DynabeadsTM purification. Abbreviations: b, beads; s, supernatant. Lane 1. WT- Biotin only. Lanes 2–3,-BirA*, lanes 4–5, BirA*-ATG8, lane 6-BirA*, lane 7, BirA*-ATG8, lane 8, BirA*, lane 9, BirA*-ATG8. Different treatments as follows: Samples 2–5, biotin was infiltrated 3 days after agroinfiltration. Samples 6–9, biotin-infiltrated concurrently with agroinfiltration buffer. Samples 6 and 7, proteins extracted 3 dpi and samples 8 and 9 were collected 4 dpi. Treatments 6 and 7 were used for the final experiment. **Figure S4.** ATG8 directly interacts with NbHYPK but not NbHYPKΔUBA and UBA-NbHYPK (Fig. 4). (a) GFP-NbHYPK, GFP-NbHYPKΔUBA, GFP-UBA-HYPK and RFP-ATG8 fusion proteins were detected in Western blot using GFP and RFP antibodies, respectively. (b) RFP-ATG8 aggregates with GFP-NbHYPK but not GFP -NbHYPKΔUBA or GFP-UBA-NbHYPK. (c) Bimolecular fluorescence complementation (BiFC) analysis showed that ATG8 was able to associate with NbHYPK but ATG8-Yn did not associate with x-Yc (plasmid without insert). NbHYPK-Yc also did not associate with x-Yn (plasmid without insert). **Figure S5.** Analysis of gene down-regulation in ATG5/7/8-VIGS plants (Fig. 5). (a) Semi-quantitative RT-PCR expression analysis of ATG8 isoforms in ATG8-silenced and non-silenced *Nicotiana benthamiana* plants. (b) qRT-PCR analysis of ATG5 and ATG7 genes in control and VIGS-plants. **Figure S6.** TMV accumulation and NbHYPK relative expression in VIGS-NbHYPK plants (Fig. 6). (a) TMV coat protein expression as detected by anti-TMV antibody. TMV coat protein is ~ 17.5 kDa. Statistical significance was determined by Student’s *t* test (*p* = .39). (b) Semi-quantitative qRT-PCR analysis of NbHYPK expression in VIGS silenced and non-silenced *Nicotiana benthamiana* plants. (ZIP 3798 kb)

Additional file 2: **Table S1.** Proteins identified through BioID (DOCX 16 kb)

Additional file 3: **Table S2.** List of primers used in this study (DOCX 18 kb)

## Data Availability

All data generated or analysed during this study are included in this published article [and its supplementary information files].

## Abbreviations

ATG8: Autophagy 8
BioID: Proximity dependent biotin identification
BirA*: Biotin protein ligase
G/Y/RFP: Green/Yellow/Red Fluorescent protein
HYPK: Huntingtin Interacting Protein K-like
MMTS: Methyl methanethiosulfonate
MS: Mass spectrometry
ROS: Reactive oxygen species
TCEP: Tris(2-carboxyethyl)phosphine
TEAB: Triethylammonium bicarbonate
TEM: Transmission electron microscope
UBA: Ubiquitin associated domain
VIGS: Virus induced gene silencing
