# Supplementary figures and images for: Proximity-dependent biotinylation screening identifies NbHYPK as a novel interacting partner of ATG8 in plants
Source: BMC Plant Biol. 2019 Jul 19;19:326. doi: 10.1186/s12870-019-1930-8 (PMC6642529; doi:10.1186/s12870-019-1930-8)

## Slide 1
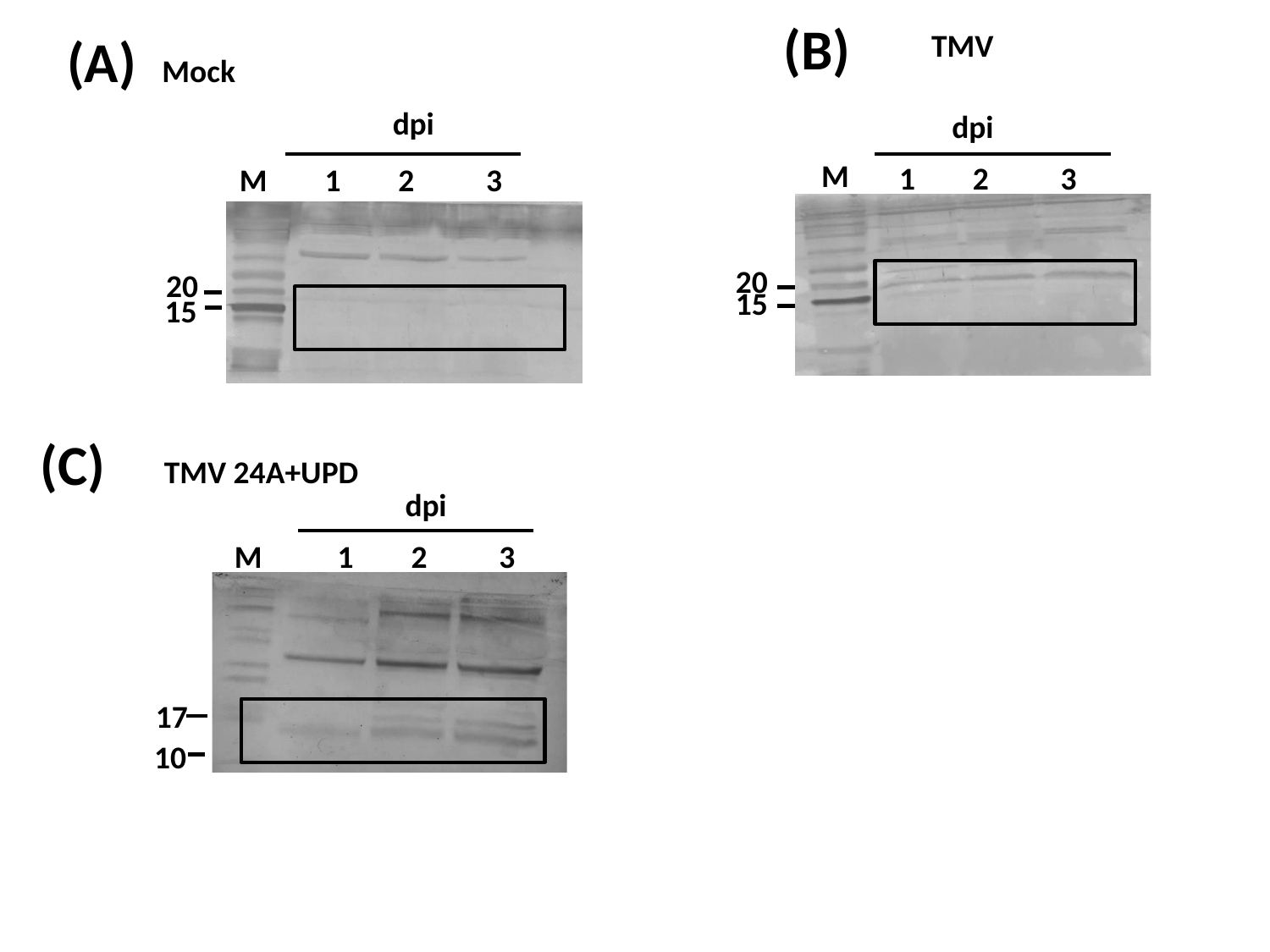

(B)
TMV
(A) Mock
dpi
dpi
M
1 2 3
M
1 2 3
20
15
20
15
(C)
TMV 24A+UPD
dpi
M
1 2 3
17
10

Supplement: Supplementary file 1 — Figure S1. Full immunoblot images for Fig. 1a using ATG8 antibody. (a) Mock. (b) TMV. (c) TMV 24A + UPD. Figure S2. Expression of BirA* and BirA_ATG8 constructs. (a) Graphical representation of control BirA*and experimental plasmids BirA*-ATG8. Full gel image for Fig. 2a carried out using anti-mCherry antiseri, for both (b) & (c) and anti-ATG8 antiserum (* indicates a weak band). (d) to ascertain fusion protein expression. (e) Detection of RFP signals from agroinfiltrated N.benthamiana leaves to confirm expression of RFP-fused BirA* and BirA*-ATG8. Figure S3. Full gel Immunoblot analysis of total biotinylated proteins for Fig. 2b and c. (a) Streptavidin-HRP blot of crude protein lysate. (b)Strep-HRP blot after DynabeadsTM purification. Abbreviations: b, beads; s, supernatant. Lane 1. WT- Biotin only. Lanes 2–3,-BirA*, lanes 4–5, BirA*-ATG8, lane 6-BirA*, lane 7, BirA*-ATG8, lane 8, BirA*, lane 9, BirA*-ATG8. Different treatments as follows: Samples 2–5, biotin was infiltrated 3 days after agroinfiltration. Samples 6–9, biotin-infiltrated concurrently with agroinfiltration buffer. Samples 6 and 7, proteins extracted 3 dpi and samples 8 and 9 were collected 4 dpi. Treatments 6 and 7 were used for the final experiment. Figure S4. ATG8 directly interacts with NbHYPK but not NbHYPKΔUBA and UBA-NbHYPK (Fig. 4). (a) GFP-NbHYPK, GFP-NbHYPKΔUBA, GFP-UBA-HYPK and RFP-ATG8 fusion proteins were detected in Western blot using GFP and RFP antibodies, respectively. (b) RFP-ATG8 aggregates with GFP-NbHYPK but not GFP -NbHYPKΔUBA or GFP-UBA-NbHYPK. (c) Bimolecular fluorescence complementation (BiFC) analysis showed that ATG8 was able to associate with NbHYPK but ATG8-Yn did not associate with x-Yc (plasmid without insert). NbHYPK-Yc also did not associate with x-Yn (plasmid without insert). Figure S5. Analysis of gene down-regulation in ATG5/7/8-VIGS plants (Fig. 5). (a) Semi-quantitative RT-PCR expression analysis of ATG8 isoforms in ATG8-silenced and non-silenced Nicotiana bentham [file 12870_2019_1930_MOESM1_ESM.zip › Supplementary Figure 1.pptx]

## Slide 1
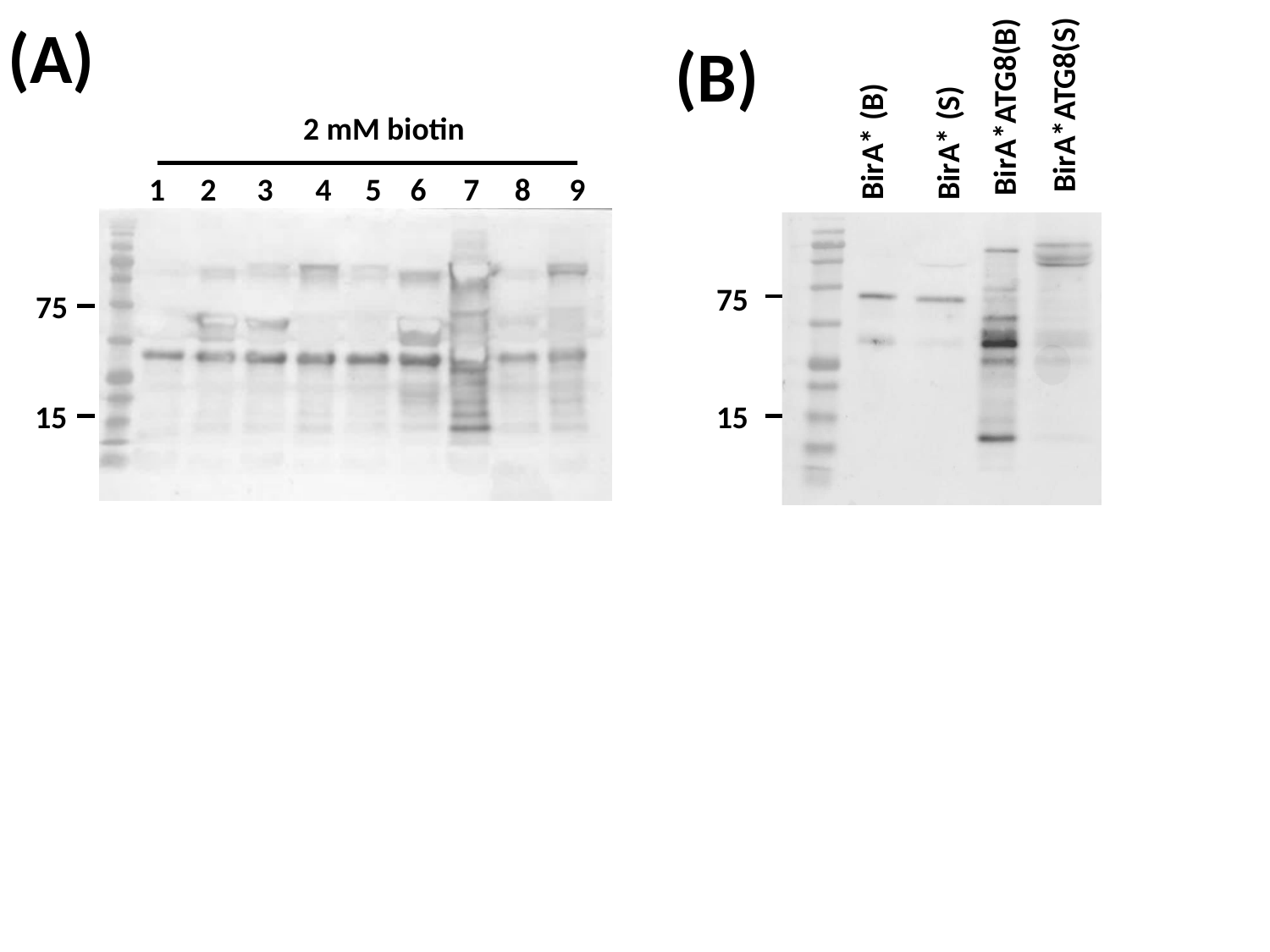

(A)
BirA*ATG8(B)
BirA*ATG8(S)
BirA* (B)
BirA* (S)
75
15
(B)
2 mM biotin
1
2
3
4
5
6
7
8
9
75
15

Supplement: Supplementary file 1 — Figure S1. Full immunoblot images for Fig. 1a using ATG8 antibody. (a) Mock. (b) TMV. (c) TMV 24A + UPD. Figure S2. Expression of BirA* and BirA_ATG8 constructs. (a) Graphical representation of control BirA*and experimental plasmids BirA*-ATG8. Full gel image for Fig. 2a carried out using anti-mCherry antiseri, for both (b) & (c) and anti-ATG8 antiserum (* indicates a weak band). (d) to ascertain fusion protein expression. (e) Detection of RFP signals from agroinfiltrated N.benthamiana leaves to confirm expression of RFP-fused BirA* and BirA*-ATG8. Figure S3. Full gel Immunoblot analysis of total biotinylated proteins for Fig. 2b and c. (a) Streptavidin-HRP blot of crude protein lysate. (b)Strep-HRP blot after DynabeadsTM purification. Abbreviations: b, beads; s, supernatant. Lane 1. WT- Biotin only. Lanes 2–3,-BirA*, lanes 4–5, BirA*-ATG8, lane 6-BirA*, lane 7, BirA*-ATG8, lane 8, BirA*, lane 9, BirA*-ATG8. Different treatments as follows: Samples 2–5, biotin was infiltrated 3 days after agroinfiltration. Samples 6–9, biotin-infiltrated concurrently with agroinfiltration buffer. Samples 6 and 7, proteins extracted 3 dpi and samples 8 and 9 were collected 4 dpi. Treatments 6 and 7 were used for the final experiment. Figure S4. ATG8 directly interacts with NbHYPK but not NbHYPKΔUBA and UBA-NbHYPK (Fig. 4). (a) GFP-NbHYPK, GFP-NbHYPKΔUBA, GFP-UBA-HYPK and RFP-ATG8 fusion proteins were detected in Western blot using GFP and RFP antibodies, respectively. (b) RFP-ATG8 aggregates with GFP-NbHYPK but not GFP -NbHYPKΔUBA or GFP-UBA-NbHYPK. (c) Bimolecular fluorescence complementation (BiFC) analysis showed that ATG8 was able to associate with NbHYPK but ATG8-Yn did not associate with x-Yc (plasmid without insert). NbHYPK-Yc also did not associate with x-Yn (plasmid without insert). Figure S5. Analysis of gene down-regulation in ATG5/7/8-VIGS plants (Fig. 5). (a) Semi-quantitative RT-PCR expression analysis of ATG8 isoforms in ATG8-silenced and non-silenced Nicotiana bentham [file 12870_2019_1930_MOESM1_ESM.zip › Supplementary Figure 3.pptx]

## Slide 1
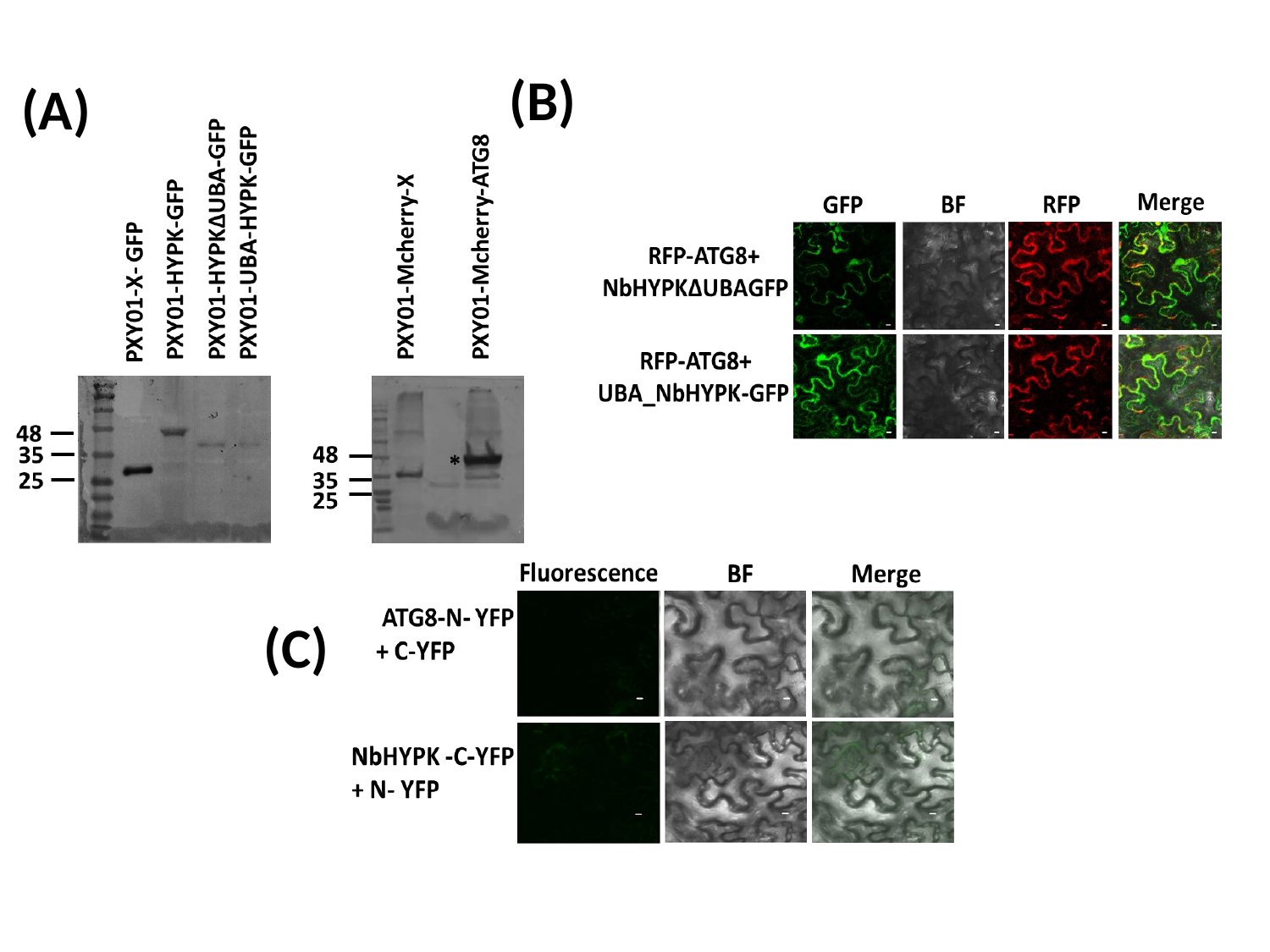

(B)
(A)
(C)

Supplement: Supplementary file 1 — Figure S1. Full immunoblot images for Fig. 1a using ATG8 antibody. (a) Mock. (b) TMV. (c) TMV 24A + UPD. Figure S2. Expression of BirA* and BirA_ATG8 constructs. (a) Graphical representation of control BirA*and experimental plasmids BirA*-ATG8. Full gel image for Fig. 2a carried out using anti-mCherry antiseri, for both (b) & (c) and anti-ATG8 antiserum (* indicates a weak band). (d) to ascertain fusion protein expression. (e) Detection of RFP signals from agroinfiltrated N.benthamiana leaves to confirm expression of RFP-fused BirA* and BirA*-ATG8. Figure S3. Full gel Immunoblot analysis of total biotinylated proteins for Fig. 2b and c. (a) Streptavidin-HRP blot of crude protein lysate. (b)Strep-HRP blot after DynabeadsTM purification. Abbreviations: b, beads; s, supernatant. Lane 1. WT- Biotin only. Lanes 2–3,-BirA*, lanes 4–5, BirA*-ATG8, lane 6-BirA*, lane 7, BirA*-ATG8, lane 8, BirA*, lane 9, BirA*-ATG8. Different treatments as follows: Samples 2–5, biotin was infiltrated 3 days after agroinfiltration. Samples 6–9, biotin-infiltrated concurrently with agroinfiltration buffer. Samples 6 and 7, proteins extracted 3 dpi and samples 8 and 9 were collected 4 dpi. Treatments 6 and 7 were used for the final experiment. Figure S4. ATG8 directly interacts with NbHYPK but not NbHYPKΔUBA and UBA-NbHYPK (Fig. 4). (a) GFP-NbHYPK, GFP-NbHYPKΔUBA, GFP-UBA-HYPK and RFP-ATG8 fusion proteins were detected in Western blot using GFP and RFP antibodies, respectively. (b) RFP-ATG8 aggregates with GFP-NbHYPK but not GFP -NbHYPKΔUBA or GFP-UBA-NbHYPK. (c) Bimolecular fluorescence complementation (BiFC) analysis showed that ATG8 was able to associate with NbHYPK but ATG8-Yn did not associate with x-Yc (plasmid without insert). NbHYPK-Yc also did not associate with x-Yn (plasmid without insert). Figure S5. Analysis of gene down-regulation in ATG5/7/8-VIGS plants (Fig. 5). (a) Semi-quantitative RT-PCR expression analysis of ATG8 isoforms in ATG8-silenced and non-silenced Nicotiana bentham [file 12870_2019_1930_MOESM1_ESM.zip › Supplementary Figure 4.pptx]

## Slide 1
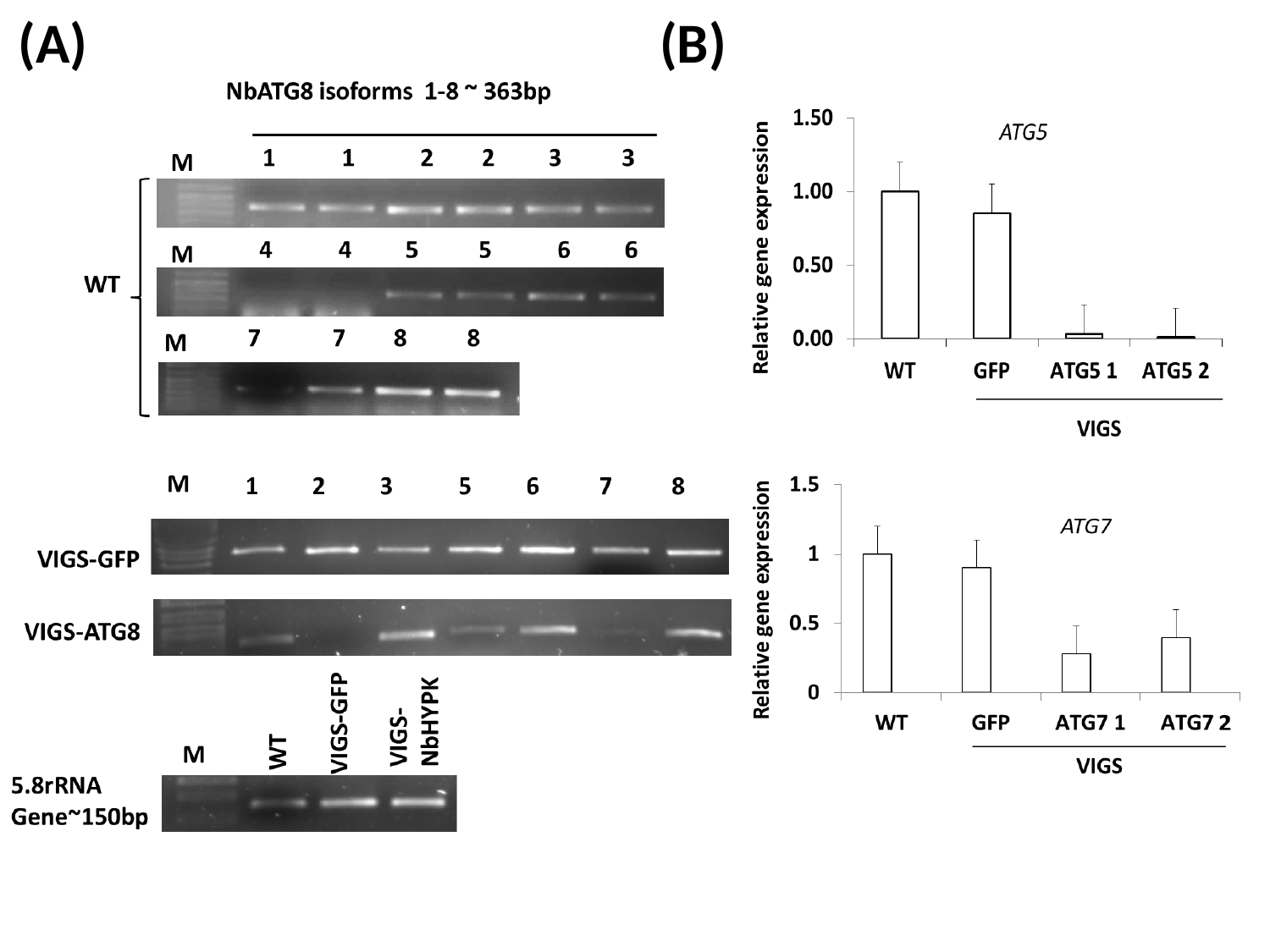

(A)
(B)

Supplement: Supplementary file 1 — Figure S1. Full immunoblot images for Fig. 1a using ATG8 antibody. (a) Mock. (b) TMV. (c) TMV 24A + UPD. Figure S2. Expression of BirA* and BirA_ATG8 constructs. (a) Graphical representation of control BirA*and experimental plasmids BirA*-ATG8. Full gel image for Fig. 2a carried out using anti-mCherry antiseri, for both (b) & (c) and anti-ATG8 antiserum (* indicates a weak band). (d) to ascertain fusion protein expression. (e) Detection of RFP signals from agroinfiltrated N.benthamiana leaves to confirm expression of RFP-fused BirA* and BirA*-ATG8. Figure S3. Full gel Immunoblot analysis of total biotinylated proteins for Fig. 2b and c. (a) Streptavidin-HRP blot of crude protein lysate. (b)Strep-HRP blot after DynabeadsTM purification. Abbreviations: b, beads; s, supernatant. Lane 1. WT- Biotin only. Lanes 2–3,-BirA*, lanes 4–5, BirA*-ATG8, lane 6-BirA*, lane 7, BirA*-ATG8, lane 8, BirA*, lane 9, BirA*-ATG8. Different treatments as follows: Samples 2–5, biotin was infiltrated 3 days after agroinfiltration. Samples 6–9, biotin-infiltrated concurrently with agroinfiltration buffer. Samples 6 and 7, proteins extracted 3 dpi and samples 8 and 9 were collected 4 dpi. Treatments 6 and 7 were used for the final experiment. Figure S4. ATG8 directly interacts with NbHYPK but not NbHYPKΔUBA and UBA-NbHYPK (Fig. 4). (a) GFP-NbHYPK, GFP-NbHYPKΔUBA, GFP-UBA-HYPK and RFP-ATG8 fusion proteins were detected in Western blot using GFP and RFP antibodies, respectively. (b) RFP-ATG8 aggregates with GFP-NbHYPK but not GFP -NbHYPKΔUBA or GFP-UBA-NbHYPK. (c) Bimolecular fluorescence complementation (BiFC) analysis showed that ATG8 was able to associate with NbHYPK but ATG8-Yn did not associate with x-Yc (plasmid without insert). NbHYPK-Yc also did not associate with x-Yn (plasmid without insert). Figure S5. Analysis of gene down-regulation in ATG5/7/8-VIGS plants (Fig. 5). (a) Semi-quantitative RT-PCR expression analysis of ATG8 isoforms in ATG8-silenced and non-silenced Nicotiana bentham [file 12870_2019_1930_MOESM1_ESM.zip › Supplementary Figure 5.pptx]

## Slide 1
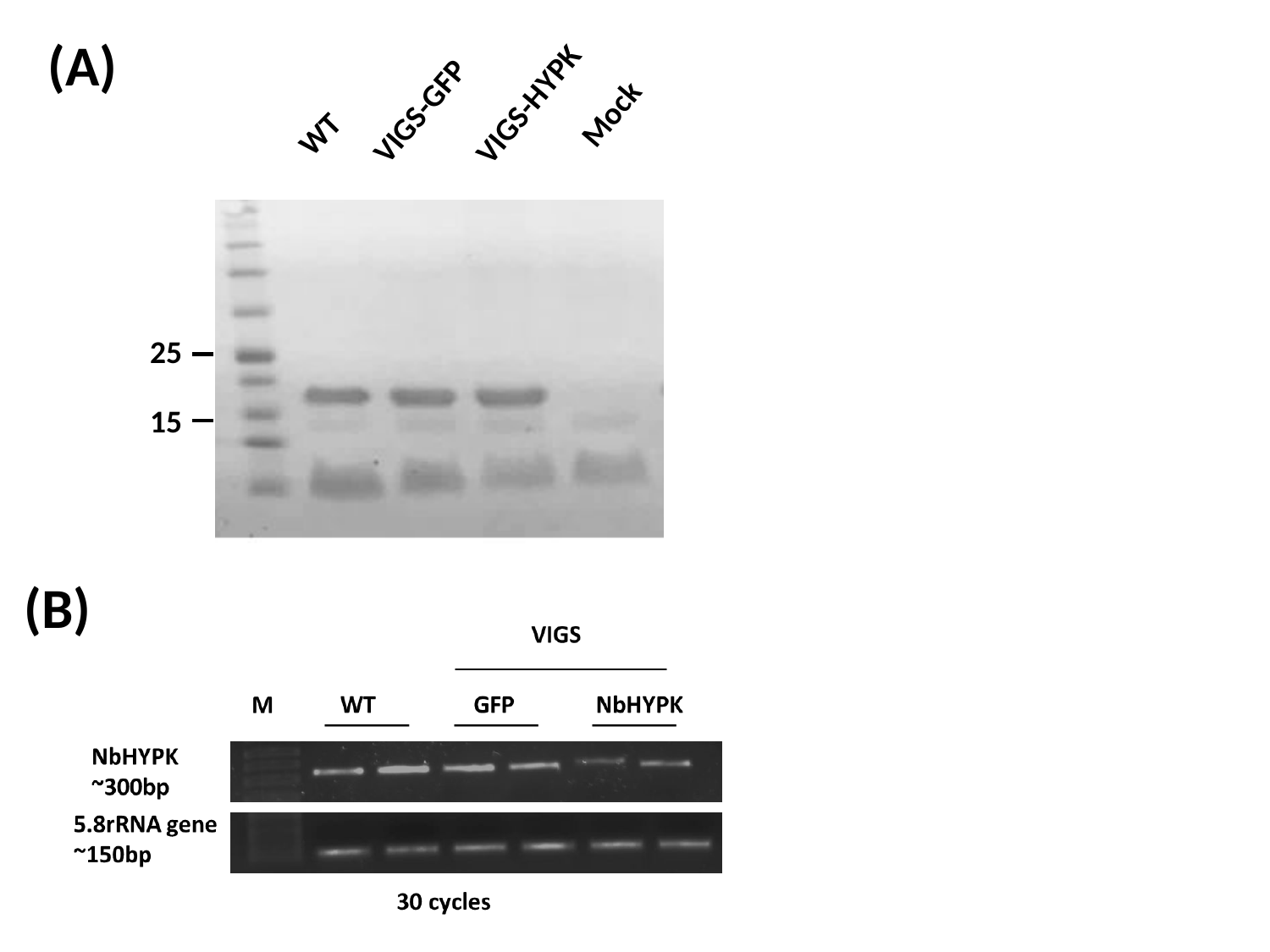

(A)
 VIGS-HYPK
 VIGS-GFP
Mock
WT
25
15
(B)

Supplement: Supplementary file 1 — Figure S1. Full immunoblot images for Fig. 1a using ATG8 antibody. (a) Mock. (b) TMV. (c) TMV 24A + UPD. Figure S2. Expression of BirA* and BirA_ATG8 constructs. (a) Graphical representation of control BirA*and experimental plasmids BirA*-ATG8. Full gel image for Fig. 2a carried out using anti-mCherry antiseri, for both (b) & (c) and anti-ATG8 antiserum (* indicates a weak band). (d) to ascertain fusion protein expression. (e) Detection of RFP signals from agroinfiltrated N.benthamiana leaves to confirm expression of RFP-fused BirA* and BirA*-ATG8. Figure S3. Full gel Immunoblot analysis of total biotinylated proteins for Fig. 2b and c. (a) Streptavidin-HRP blot of crude protein lysate. (b)Strep-HRP blot after DynabeadsTM purification. Abbreviations: b, beads; s, supernatant. Lane 1. WT- Biotin only. Lanes 2–3,-BirA*, lanes 4–5, BirA*-ATG8, lane 6-BirA*, lane 7, BirA*-ATG8, lane 8, BirA*, lane 9, BirA*-ATG8. Different treatments as follows: Samples 2–5, biotin was infiltrated 3 days after agroinfiltration. Samples 6–9, biotin-infiltrated concurrently with agroinfiltration buffer. Samples 6 and 7, proteins extracted 3 dpi and samples 8 and 9 were collected 4 dpi. Treatments 6 and 7 were used for the final experiment. Figure S4. ATG8 directly interacts with NbHYPK but not NbHYPKΔUBA and UBA-NbHYPK (Fig. 4). (a) GFP-NbHYPK, GFP-NbHYPKΔUBA, GFP-UBA-HYPK and RFP-ATG8 fusion proteins were detected in Western blot using GFP and RFP antibodies, respectively. (b) RFP-ATG8 aggregates with GFP-NbHYPK but not GFP -NbHYPKΔUBA or GFP-UBA-NbHYPK. (c) Bimolecular fluorescence complementation (BiFC) analysis showed that ATG8 was able to associate with NbHYPK but ATG8-Yn did not associate with x-Yc (plasmid without insert). NbHYPK-Yc also did not associate with x-Yn (plasmid without insert). Figure S5. Analysis of gene down-regulation in ATG5/7/8-VIGS plants (Fig. 5). (a) Semi-quantitative RT-PCR expression analysis of ATG8 isoforms in ATG8-silenced and non-silenced Nicotiana bentham [file 12870_2019_1930_MOESM1_ESM.zip › Supplementary Figure 6.pptx]
