# Supplementary material for: Proximity-dependent biotinylation screening identifies NbHYPK as a novel interacting partner of ATG8 in plants
Source: BMC Plant Biol. 2019 Jul 19;19:326. doi: 10.1186/s12870-019-1930-8 (PMC6642529; doi:10.1186/s12870-019-1930-8)
Supplement: Supplementary file 2 — Table S1. Proteins identified through BioID (DOCX 16 kb) [file 12870_2019_1930_MOESM2_ESM.docx]

| **No.** | **Uniprot Accession No.** | **Protein Name** |
| --- | --- | --- |
|  | A0A1J6K458 | Web family protein, chloroplastic |
|  | A0A1U7VRP7 | Titin |
|  | A0A1U7XVB2 | Eukaryotic translation initiation factor 3 (eIF3) subunit J |
|  | A0A1U7YE93 | Glycine cleavage system P protein |
|  | A0A1U7X7A4 | Glycerate dehydrogenase |
|  | C3RXI5 | Plastid transketolase |
|  | A0A1U7WQQ4 | Glucan endo-1,3-beta-glucosidase, acidic isoform GI9-like |
|  | A0A1U7V0I2 | Stress protein DDR48-like |
|  | A0A1S3YK01 | Protein TSS isoform X3 |
|  | A0A1U7WJE7 | DnaJ homolog subfamily C member 2 (DNAJC2)-like |
|  | Q6QND3 | Putative pyridoxine biosynthesis protein isoform A |
|  | A0A1U7Y895 | Serine/threonine-protein phosphatase 6 (PP6) regulatory subunit 3-like isoform X4 |
|  | A0A1U7VJZ0 | Serine/threonine-protein kinase pakA-like |
|  | S6A7M3 | 5-methyltetrahydropteroyltriglutamate--homocysteine methyltransferase 1 |
|  | A0A1U7Z0Z6 | Phototropin-2 (PHOT2) |
|  | Q84QE8 | Oxygen evolving complex (OEC) 33 kDa photosystem II protein |
|  | Q40540 | Pit2 protein |
|  | G3LUZ7 | Photosystem I P700 chlorophyll a apoprotein A2 (psaB) |
|  | X5JM82 | Ankyrin repeat domain containing protein |
|  | A0A1U7X1U7 | Chloroplast stem-loop binding protein of 41 kDa b, chloroplastic (CSP41b) |
|  | A0A1J6IH70 | 4-Coumarate--CoA ligase-like 6 |
|  | A0A1U7USU9 | Proton pump-interactor 1 (PPI1)-like isoform X1 |
|  | A0A1J6JP27 | Translocase of chloroplast 159 (Toc159), chloroplastic |
|  | A0A1S4CU65 | Uncharacterized protein At5g39570-like |
|  | A0A1J6KEX0 | Uncharacterized protein |
|  | A0A1J6IRF3 | Uncharacterized protein |
|  | A0A1U7V0N4 | Uncharacterized protein LOC104213946 |
|  | A0A1U7YKY0 | Uncharacterized protein LOC104245530 |
|  | A0A1U7XLG9 | Uncharacterized protein LOC104235688 |
|  | A0A1U7X805 | Uncharacterized protein LOC104231332 isoform X2 |
|  | A0A1U7WTE3 | Uncharacterized protein LOC104231829 |
|  | A0A1U7V5R4 | uncharacterized protein LOC104209985 isoform X2 |
|  | A0A1J6L2Q2 | Uncharacterized protein |
|  | A0A1U7X7S8 | Uncharacterized protein LOC104231263 isoform X1 |
|  | A0A1U7YW82 | Uncharacterized abhydrolase domain-containing protein DDB_G0269086-like |
|  | A0A1S3ZK36 | Uncharacterized protein OsI_027940-like isoform X2 |
|  | A0A1U7W5E2 | Uncharacterized protein OsI_027940-like isoform X2 |
