# Supplementary material for: Proximity-dependent biotinylation screening identifies NbHYPK as a novel interacting partner of ATG8 in plants
Source: BMC Plant Biol. 2019 Jul 19;19:326. doi: 10.1186/s12870-019-1930-8 (PMC6642529; doi:10.1186/s12870-019-1930-8)
Supplement: Supplementary file 3 — Table S2. List of primers used in this study (DOCX 18 kb) [file 12870_2019_1930_MOESM3_ESM.docx]

| **Experiment** | **Primers** | **Sequence (5’ - 3’)** |
| --- | --- | --- |
| **ATG8 gene &**  **Subcellular localization** | ATG8aF | CCCGGGATGGCCAAAAGCTCCTTCAAATTG |
|  | ATG8aR | GGATCCTTAGAACGATCCGAATGTATTCTCTCCAC |
| **Yeast two hybrid (ATG8)** | GA_BD_ATG8F | acctgcatatggccatggaggccgtt ttATGGCCAAGAGTTCTTTCAAGC |
|  | GA_BD_ATG8R | tcccgtatcgatgcccacccgggtggaattTTAATTTCCAAGCTCAAGGAAC |
| **VIGS (Autophagy)** | ATG5F | GGTACCATGGGAAGTAAAGGGGCAGGA, |
|  | ATG5R | CCCGGGACGTTCAGGTTCTGCACAAAGA |
|  | ATG7F | GGTACCTTGTCTGATTTGATTAATTCACATGA |
|  | ATG7R | CCCGGGATCAATTGGTGCAACCAC; |
|  | ATG8F | GGTACCTTA TGG CCA AGA GTT CTT TCA AGC |
|  | ATG8R | CCCGGGTTA ATT TCC AAG CTC AAG GAA CCC |
| **BioID (HA & BirA*)** | BirA*_F | TCTAGATACCCATACGACGTACCAGATTACGCTGACAAGGACAACACCGTGCCC |
|  | BirA*_R | CCCGGGCTTCTCTGCGCTTCTCAGGGAGATTT |
| **HYPK-Like protein** | HYPK-F | atggaagctggagatgaagcg |
|  | HYPK-R | ttaattcagcaaatgccttattgcagc |
| **Yeast two Hybrid (HYPK)** | GA_AD_HYPK F | attacgctcatatggccatggaggccagtgTTatggaagctggagatga |
|  | GA_AD_HYPK R | gatgcccacccgggtggaattttaattcagcaaatgccttattgcagct |
|  | NbHYPKΔUBA F | atggaagctggagatgaagcg |
|  |  |  |
|  | NbHYPKΔUBA R: | TTAagcagctagttccttttccctcat |
|  | GA_ AD_NbHYPKΔUBA F: | attacgctcatatggccatggaggccagtgTTatggaagctggagatga |
|  | GA_ AD_NbHYPKΔUBA R: | cgatgcccacccgggtggaattTTAagcagctagttccttttcc |
|  | NbHYPK_UBA F: | ATGgttaagatcaatgctgctga |
|  | NbHYPK_UBA R: | TTAcagcaaatgccttattgcagc |
|  | GA_ AD_NbHYPK_UBA F: | tcatatggccatggaggccagtgTTATGgttaagatcaatgctgct |
|  | GA_ AD_NbHYPK_UBA R: | tgcccacccgggtggaattTTAcagcaaatgccttattgcagctac |
| **VIGS (HYPK)** | VIGS_HYPK F: | tagagatggtggactcaaaggattt |
|  | VIGS_HYPK R: | gcaaatgccttattgcagctaca |
|  | GA_CD3_HYPK F: | CTGGTTACTGTATCACTTACCCGAGTT tagagatggtggactcaaaggattt |
|  | GA_CD3_HYPKR: | TAGTTTAATGTCTTCGGGACATGCCCgcaaatgccttattgcagctacagcat |
| **BiFC** | GA_NYFP_ATG8F | CTC CGG ACT CAG ATC TCG AGC TCA TAT GGC CAA GAG TTC TTT CAA GCT TGA A |
|  | GA_NYFP_ATG8R | CCC GCG GTA CCG TCG ACT GCA GAA TTT TAA TTT CCA AGC TCA AGG AAC CC |
|  | GA_CYFP_HYPKF: | TCC GGA CTC AGA TCT CGA GCT CAT ATG GAA GCT GGA GAT GAA GCG CTA G |
|  | GA_CYFP_HYPKR | CGG TAC CGT CGA CTG CAG AAT TTT AAT TCA GCA AAT GCC TTA TTG CAG CTA |
|  | GA_CYFP__HYPKΔUBA F | TCAGATCTCGAGCTCATatggaagctggagatgaagcgctagaga |
|  | GA_CYFP__HYPKΔUBA F | GCCCGCGGTACCGTCGACTGCAGAATTTTAagcagctagttccttttc |
|  | GA_CYFP_ NbHYPK_UBA F | GACTCAGATCTCGAGCTCATATGgttaagatcaatgctgctgatatt |
|  | GA_CYFP_ NbHYPK_UBA R | CGCGGTACCGTCGACTGCAGAATTTTAcagcaaatgccttatt |
| **Subcellular localization (HYPK)** | GA_HYPK_GFP F | TTG GAG AGG ACA GCC CAG ATC AAT GGA AGC TGG AGA TGA AGC G |
|  | GA_HYPK_GFP R | CAG AGC CAC CTC CCT CGA GAC CAT TCA GCA AAT GCC TTA TTG CA |
|  | GA_ΔUBAHYPK_GFP F | TGGAGAGGACAGCCCAGATCAatggaagctggagatgaagcgctag |
|  | GA_ΔUBAHYPK_GFP R | AGAGCCACCTCCCTCGAGACCagcagctagttccttttccctcatc |
|  | GA_HYPK_UBA_GFP F | AGAGGACAGCCCAGATCAATGgttaagatcaatgctgctgatattgatata |
|  | GA_HYPK_UBA_GFP R | CCACCTCCCTCGAGACCcagcaaatgccttattgcagc |
